# Comparison of inspiratory and expiratory airway volumes and luminal areas among standing, sitting, and supine positions using upright and conventional CT

**DOI:** 10.1038/s41598-022-25865-0

**Published:** 2022-12-09

**Authors:** Yoshitake Yamada, Minoru Yamada, Shotaro Chubachi, Yoichi Yokoyama, Shiho Matsuoka, Akiko Tanabe, Yuki Niijima, Mitsuru Murata, Takayuki Abe, Koichi Fukunaga, Masahiro Jinzaki

**Affiliations:** 1 Department of Radiology, Keio University School of Medicine, 35 Shinanomachi, Shinjuku-Ku, Tokyo, 160-8582 Japan; 2 Division of Pulmonary Medicine, Department of Medicine, Keio University School of Medicine, 35 Shinanomachi, Shinjuku-Ku, Tokyo, 160-8582 Japan; 3 Department of Clinical Laboratory, Keio University Hospital, 35 Shinanomachi, Shinjuku-Ku, Tokyo, 160-8582 Japan; 4 Office of Radiation Technology, Keio University Hospital, 35 Shinanomachi, Shinjuku-Ku, Tokyo, 160-8582 Japan; 5 Department of Laboratory Medicine, Keio University School of Medicine, 35 Shinanomachi, Shinjuku-Ku, Tokyo, 160-8582 Japan; 6 School of Data Science, Yokohama City University, 22-2 Seto, Kanazawa-Ku, Yokohama, Kanagawa 236-0027 Japan; 7 Biostatistics, Clinical and Translational Research Center, Keio University School of Medicine, 35 Shinanomachi, Shinjuku-Ku, Tokyo, 160-8582 Japan

**Keywords:** Anatomy, Medical research, Respiration, Imaging, X-ray tomography

## Abstract

Upright computed tomography (CT) provides physiologically relevant images of daily life postures (sitting and standing). The volume of the human airway in sitting or standing positions remains unclear, and no clinical study to date has compared the inspiratory and expiratory airway volumes and luminal areas among standing, sitting, and supine positions. In this prospective study, 100 asymptomatic volunteers underwent both upright (sitting and standing positions) and conventional (supine position) CT during inspiration and expiration breath-holds and the pulmonary function test (PFT) within 2 h of CT. We compared the inspiratory/expiratory airway volumes and luminal areas on CT among the three positions and evaluated the correlation between airway volumes in each position on CT and PFT measurements. The inspiratory and expiratory airway volumes were significantly higher in the sitting and standing positions than in the supine position (inspiratory, 4.6% and 2.5% increase, respectively; expiratory, 14.9% and 13.4% increase, respectively; all *P* < 0.001). The inspiratory and expiratory luminal areas of the trachea, bilateral main bronchi, and average third-generation airway were significantly higher in the sitting and standing positions than in the supine position (inspiratory, 4.2‒10.3% increases, all *P* < 0.001; expiratory, 6.4‒12.8% increases, all *P* < 0.0001). These results could provide important clues regarding the pathogenesis of orthopnea. Spearman’s correlation coefficients between the inspiratory airway volume on CT and forced vital capacity and forced expiratory volume in 1 s on PFT were numerically higher in the standing position than in the supine position (0.673 vs. 0.659 and 0.669 vs. 0.643, respectively); however, no statistically significant differences were found. Thus, the airway volumes on upright and conventional supine CT were moderately correlated with the PFT measurements.

## Introduction

Humans are in an upright (sitting or standing) position during daytime hours; however, most of the 3-dimensional diagnostic imaging techniques, such as magnetic resonance imaging or computed tomography (CT), are performed in a supine position. Thus, the volume of the human airway in the sitting or standing position is still unclear. Chest radiography is the most common imaging examination performed in the upright position^1^; however, chest radiography provides 2-dimensional images that do not accurately depict the airway volume.

An upright 320-detector-row CT scanner has recently been developed to assess the 3-dimensional anatomy of a human in the upright position^2^. This upright CT scanner provides physiologically relevant images of daily life postures, such as sitting and standing positions, and enables the acquisition of volume data of the entire chest (isotropic 0.5-mm voxel size) in about 5 seconds^3,4^. A previous study compared the inspiratory and expiratory lung and lobe volumes among standing, sitting, and supine positions^3^. However, to the best of our knowledge, no clinical study to date has compared both the inspiratory and expiratory airway volumes and luminal areas among the three positions. We hypothesized that the inspiratory and expiratory airway volumes and luminal areas will be different between the upright (sitting and standing) and supine positions because of the different directions of gravity in relation to the chest in these positions. We also hypothesized that upright CT airway volume will be more strongly associated than supine CT with the measurements on the pulmonary function test (PFT) because the PFT is performed in the upright position.

The purpose of this study was to compare the inspiratory and expiratory airway volumes and luminal areas on CT among the standing, sitting, and supine positions and to determine the correlation between the airway volumes in each position on CT and measurements on the PFT.

## Methods

### Study population

This prospective study was approved by the Keio University School of Medicine Ethics Committee (No. 20160384). All participants provided written informed consent (UMIN Clinical Trials Registry [UMIN-CTR]: UMIN000026586). All methods were performed in accordance with the relevant guidelines and regulations. In addition, informed consent was obtained to publish the images in an online open access publication. From June 2017 to August 2018, 100 asymptomatic volunteers from a volunteer recruitment company were enrolled in this study. To ensure that normal whole-body anatomy was evaluated, volunteers with a history of diabetes, dyslipidemia, hypertension, dysuria, and smoking; those who had any type of symptoms; those who were pregnant or possibly pregnant; and those who were currently undergoing treatment or had undergone surgery were excluded from the study. The data of the 100 included volunteers had been analyzed for different purposes in a previous study that evaluated lung and lobe volumes^3^ but not airway volume.

### CT imaging protocol

**Figure 1:**
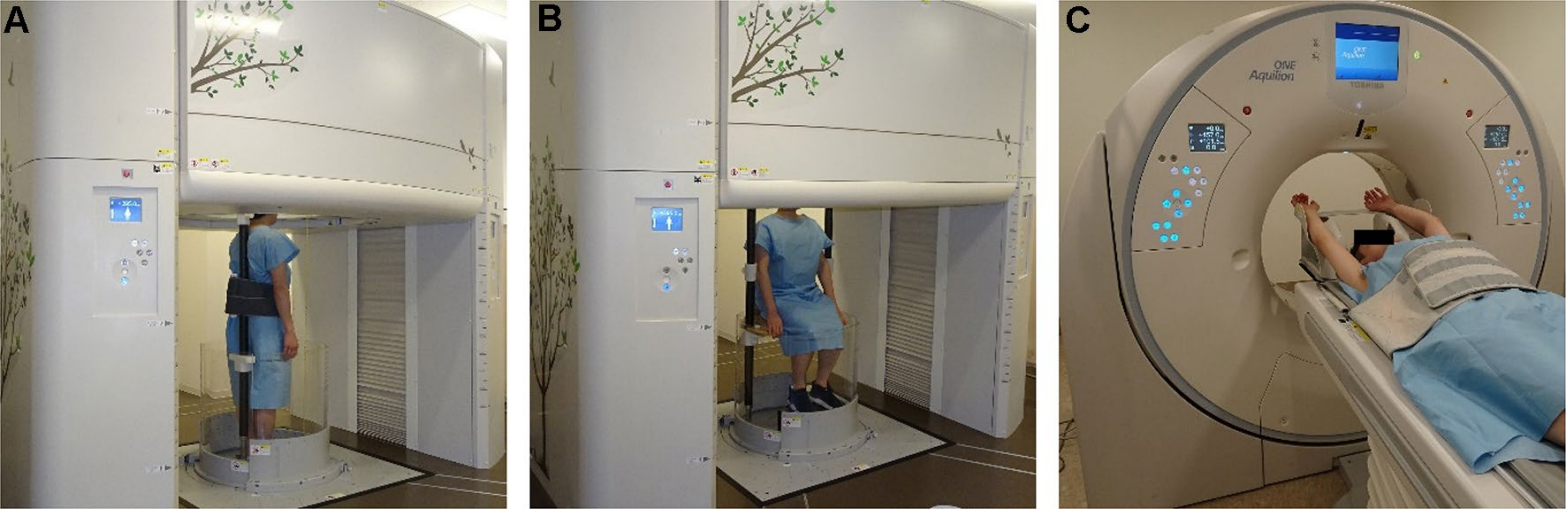
Upright CT examination in the standing position (**A**), upright CT examination in the sitting position (**B**), and conventional CT examination in the supine position (**C**). Upright CT examinations in the standing position (**A**) and sitting position (**B**) were performed with the subject’s arms down during both deep inspiration breath-hold and expiration breath-hold. Conventional CT in the supine position (**C**) was performed with the subject’s arms raised during both deep inspiration breath-hold and expiration breath-hold. *CT* computed tomography.

All participants underwent both upright body trunk low-radiation-dose CT in standing (Fig. 1A) and sitting positions with arms down at their sides (Fig. 1B), performed using an upright 320-detector-row CT (prototype TSX-401R, Canon Medical Systems, Otawara, Japan)^2–4^, and conventional body trunk low-radiation-dose CT in the supine position with arms raised (Fig. 1C), performed using a 320-detector-row CT (Aquilion ONE, Canon Medical Systems), within 2 h on the same day. The participants were scanned in the three positions during both deep-inspiration breath-hold and expiration breath-hold (at the end-tidal expiration, near functional residual capacity on PFT), as described in previous studies^3,5^. The order of standing, sitting and supine CT was not randomized.

All CT examinations were unenhanced and performed with automatic exposure control using a noise index of 24 for a slice thickness of 5 mm (tube current range, 10–350 mA)^3^. Other scanning parameters were the same for standing, sitting, and supine CT scans: peak tube voltage, 100 kVp; rotation speed, 0.5 s; slice collimation, 0.5 mm × 80; and pitch factor, 0.813^3^. The series of contiguous 0.5-mm-thick images was reconstructed with Adaptive Iterative Dose Reduction 3D (Canon Medical Systems)^6^.

### Pulmonary function test

All participants underwent PFTs within 2 h of CT examinations on the same day. The PFT was performed with the participants in a stable condition while sitting, using a spirometer (Chestac-8900, Chest M.I., Tokyo, Japan) in accordance with ATS/European Respiratory Society recommendations^7,8^. The total lung capacity and residual volume were measured using the multi-breath helium dilution method. The predicted values of the spirometric measurements were derived from the guidelines of the Japanese Respiratory Society^9^.

### Airway volume measurements on CT

**Figure 2:**
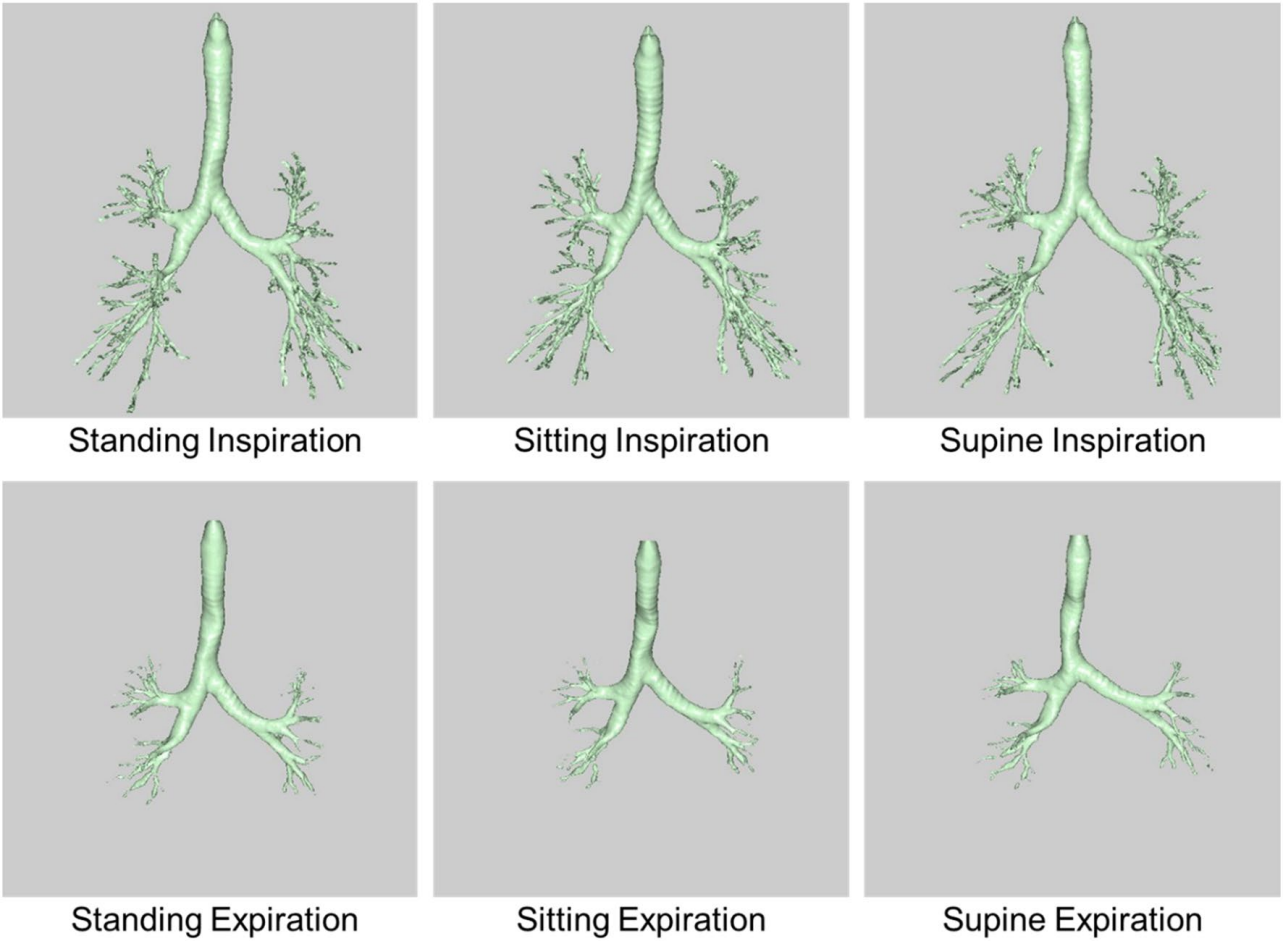
Representative volume rendering airway images in a 39-year-old man acquired in the standing, sitting, and supine positions.

Airway volume measurements for all 100 volunteers in the three positions were performed by two radiologists, in consensus, with 15 (Y.YA.) and 7 (Y.YO.) years of experience using a commercially available workstation (Synapse Vincent, Fuji Film Co., Ltd., Tokyo, Japan)^3,4,10–15^. This workstation incorporates a computer-aided detection system and automatically extracts the entire airway tree (from the trachea to all bilateral airways with lumen diameters of more than approximately 1.5 mm), which was defined as the airway volume (Fig. 2)^3,4,10–15^. In addition, all branches of the third- to sixth-generation airways in all segments were manually identified by tracking from the third to sixth generation^13,15,16^. The cross-sectional images perpendicular to the longitudinal center line of the lumen were generated for each branch, and the luminal areas in the middle-third portion were automatically measured and averaged^10,13,15^. The mean luminal area for each generation airway was calculated in all segments^10,15^. All measurements were performed in a blinded and randomized manner. During all the measurements, the radiologists were also blinded to patient characteristics and PFT results. The airway volume changes from expiration to inspiration on CT, and the ratio of inspiratory airway volume to expiratory airway volume were calculated.

### Statistical analysis

The data are presented as mean ± standard deviation. A paired *t* test was performed to analyze the differences in the airway volumes and luminal areas among standing, sitting, and supine positions; the differences in the airway volume changes from expiration to inspiration among the three positions; and the differences in the ratio of inspiratory airway volume to expiratory airway volume among the three positions. Bonferroni correction was used for multiple comparisons. The difference in age between women and men was assessed using Student’s *t* test. The associations between the airway volumes on CT in each position and the measurements on the PFT were evaluated using Spearman’s rank correlation test. Paired correlation coefficients (PFT measurements vs. airway volumes on CT among the three positions) were compared using a mixed-effect model with Bonferroni correction for multiple comparisons (e.g. across all PFT measurements and three positions). The significance level for all tests was 5% (two-sided). All data were analyzed using a commercially available software program (JMP version 12; SAS Institute Inc, Cary, NC, USA).

## Results

### Participant characteristics

The clinical characteristics of all the participants (n = 100) are shown in Table 1. No significant difference was found in age between the men and women (44.9 ± 10.0 years vs. 48.0 ± 11.9 years, *P* = 0.210).

**Table 1:** Characteristics of the study population (100 volunteers).

Demographic variables	Value	
	Mean ± SD or n	Range
Age (years)	47.1 ± 11.4	30‒79
Sex (female/male) (n)	69/31	
Height (cm)	161.2 ± 9.0	141.4‒187.5
Weight (kg)	57.6 ± 12.1	37.8‒106.8
Body mass index (kg/m^2^)	22.1 ± 3.6	15.7‒33.7
**Pulmonary function test**		
VC (L)	3.63 ± 0.91	1.79‒6.13
FVC (L)	3.56 ± 0.91	1.79‒5.98
VC (% predicted)	105.4 ± 10.4	81.6‒138.9
FEV1 (L)	2.86 ± 0.74	1.37‒4.61
FEV1 (% predicted)	101.9 ± 12.2	71.1‒133.5
FEV1/FVC (%)	80.6 ± 7.5	55.5‒96.0
Tidal volume (L)	0.57 ± 0.17	0.29‒1.06
Residual volume (L)	1.51 ± 0.38	0.80‒2.92
Functional residual capacity (L)	2.91 ± 0.70	1.36‒4.86
Total lung capacity (L)	5.13 ± 1.17	2.65‒8.22

*SD* standard deviation, *VC* vital capacity, *FVC* forced vital capacity, *FEV1* forced expiratory volume in 1 s.

### Airway volumes on CT in standing, sitting, and supine positions

The inspiratory airway volumes were significantly higher in the sitting and standing positions than in the supine position (4.6% and 2.5% increase, respectively; both *P* < 0.004) (Table 2). The expiratory airway volumes were significantly higher in the sitting and standing positions than in the supine position (14.9% and 13.4% increase, respectively; both *P* < 0.0001). The inspiratory airway volumes were significantly higher in the sitting position than in the standing position (2.0%; *P* = 0.0072). No significant difference was found in the expiratory airway volumes between the sitting and standing positions (*P* = 0.0898).

**Table 2:** Inspiratory and expiratory airway volumes on CT in standing, sitting, and supine positions (100 volunteers).

	Airway volume (mL) average ± standard deviation (range)			P value			Average percent increase/decrease in volume in standing position compared with that in supine position	Average percent increase/decrease in volume in sitting position compared with that in supine position	Average percent increase/decrease in volume in sitting position compared with that in standing position
	Standing	Sitting	Supine	Supine vs. Standing	Supine vs. Sitting	Standing vs. Sitting			
Inspiratory airway volume	60.4 ± 16.9 (34.7‒100.7)	61.6 ± 17.7 (31.5‒105.1)	58.9 ± 17.8 (20.8‒98.3)	0.0039	< 0.0001	0.0072	+ 2.5%	+ 4.6%	+ 2.0%
Expiratory airway volume	44.0 ± 13.3 (17.2‒80.0)	44.6 ± 13.2 (20.4‒78.8)	38.8 ± 11.1 (16.2‒69.6)	< 0.0001	< 0.0001	0.0898	+ 13.4%	+ 14.9%	+ 1.4%

*CT* computed tomography.

*P* < 0.0167 was considered to be statistically significant, with Bonferroni correction for multiple comparisons.

### Airway volume changes from expiration to inspiration on CT in standing, sitting, and supine positions

The airway volume changes from expiration to inspiration in the sitting and standing positions were significantly lower than those in the supine position (17.0 ± 7.8 and 16.3 ± 7.3 mL, respectively vs. 20.1 ± 10.4 mL; both *P* < 0.0001). No significant difference was found in the airway volume change from expiration to inspiration between the sitting and standing positions (*P* = 0.2181).

### Ratio of inspiratory airway volume to expiratory airway volume in standing, sitting, and supine positions

The ratios of inspiratory airway volumes to expiratory airway volumes in the sitting and standing positions were significantly lower than those in the supine position (1.40 ± 0.18 and 1.39 ± 0.19, respectively vs. 1.53 ± 0.25; both *P* < 0.0001). No significant difference was found in the ratio of inspiratory airway volume to expiratory airway volume between the sitting and standing positions (*P* = 0.9359).

### Airway luminal areas on CT in standing, sitting, and supine positions

The inspiratory airway luminal areas of the trachea, bilateral main bronchi, and average third-, fourth-, fifth-, and sixth-generation airway were significantly higher in the sitting and standing positions than those in the supine position (4.2‒11.0% increases, all *P* < 0.006) (Table 3). The expiratory luminal areas of the trachea, bilateral main bronchi, and average third-generation airway were significantly higher in the sitting and standing positions than those in the supine position (6.4‒12.8% increases, all *P* < 0.0001) (Table 3). No significant differences were found in the inspiratory or expiratory airway luminal areas of the trachea, bilateral main bronchi, or average third-, fourth-, fifth-, or sixth-generation airways between the sitting and standing positions (Table 3).

**Table 3:** Inspiratory and expiratory airway luminal areas on CT in standing, sitting, and supine positions (100 volunteers).

	Airway luminal area (mm^2^) average ± standard deviation (range)			P value			Average percent increase/decrease in area in standing position compared with that in supine position	Average percent increase/decrease in area in sitting position compared with that in supine position	Average percent increase/decrease in area in sitting position compared with that in standing position
	Standing	Sitting	Supine	Supine vs. Standing	Supine vs. Sitting	Standing vs. Sitting			
Trachea (Inspiratory)	254.4 ± 71.5 (139.9‒456.5)	253.5 ± 73.0 (132.8‒457.4)	243.2 ± 70.7 (111.7‒434.3)	< 0.0001	< 0.0001	0.5677	+ 4.6%	+ 4.2%	-0.4%
Right main bronchus (Inspiratory)	191.8 ± 59.3 (102.0‒406.4)	193.5 ± 60.0 (104.3‒387.7)	180.7 ± 53.9 (94.3‒297.5)	0.0004	< 0.0001	0.5255	+ 6.1%	+ 7.1%	+ 0.9%
Left main bronchus (Inspiratory)	115.8 ± 32.7 (53.1‒216.8)	115.3 ± 31.4 (49.1‒210.6)	105.9 ± 29.8 (42.6‒201.6)	< 0.0001	< 0.0001	0.7943	+ 9.3%	+ 8.9%	-0.4%
Average third-generation airway (Inspiratory)	88.7 ± 26.7 (46.3‒211.5)	90.8 ± 27.3 (46.4‒205.1)	82.3 ± 24.8 (37.6‒158.48)	< 0.0001	< 0.0001	0.0827	+ 7.8%	+ 10.3%	+ 2.4%
Average fourth-generation airway (Inspiratory)	49.0 ± 15.2 (23.2‒104.0)	50.2 ± 15.7 (22.6‒98.0)	46.6 ± 13.7 (19.3‒83.2)	0.0056	< 0.0001	0.1442	+ 5.2%	+ 7.7%	+ 2.4%
Average fifth-generation airway (Inspiratory)	28.4 ± 8.5 (13.8‒66.2)	29.2 ± 9.1 (13.4‒59.7)	26.3 ± 7.5 (10.5‒42.3)	0.0002	< 0.0001	0.1427	+ 8.0%	+ 11.0%	+ 2.8%
Average sixth-generation airway (Inspiratory)	16.2 ± 4.5 (7.6‒27.8)	16.1 ± 5.1 (8.3‒32.1)	14.6 ± 4.8 (6.1‒27.6)	0.0003	0.0002	0.8304	+ 11.0%	10.2%	-0.6%
Trachea (Expiratory)	221.4 ± 62.3 (104.5‒384.2)	220.9 ± 60.5 (115.3‒376.1)	207.7 ± 55.5 (105.8‒372.9)	< 0.0001	< 0.0001	0.6004	+ 6.6%	+ 6.4%	-0.2%
Right main bronchus (Expiratory)	164.1 ± 53.6 (32.7‒302.6)	167.2 ± 52.1 (77.7‒359.0)	149.3 ± 47.9 (36.8‒271.1)	< 0.0001	< 0.0001	0.1522	+ 9.9%	+ 12.0%	+ 1.9%
Left main bronchus (Expiratory)	99.6 ± 27.4 (33.7‒171.3)	100.5 ± 27.5 (50.5‒182.2)	89.1 ± 24.7 (41.8‒159.9)	< 0.0001	< 0.0001	0.0825	+ 11.8%	+ 12.8%	+ 0.9%
Average third-generation airways (Expiratory)	71.7 ± 23.4 (10.4‒175.6)	73.2 ± 22.9 (36.0‒183.2)	66.4 ± 21.7 (25.1‒152.0)	< 0.0001	< 0.0001	0.0349	+ 8.0%	+ 10.2%	+ 2.1%
Average fourth-generation airways (Expiratory)	37.2 ± 12.6 (4.6‒86.4)	37.6 ± 12.6 (13.6‒91.7)	35.7 ± 11.1 (14.9‒67.3)	0.0881	0.0038	0.3832	+ 4.2%	+ 5.3%	+ 1.1%
Average fifth-generation airways (Expiratory)	19.6 ± 7.5 (2.1‒52.9)	20.3 ± 8.3 (1.7‒69.3)	18.9 ± 6.0 (8.2‒33.4)	0.3377	0.0269	0.1276	+ 3.7%	+ 7.4%	+ 3.6%
Average sixth-generation airway (Expiratory)	11.3 ± 4.3 (2.7‒27.6)	11.5 ± 4.2 (3.8‒27.6)	10.4 ± 3.3 (2.1‒20.37)	0.0385	0.0040	0.3630	+ 8.7%	+ 10.6%	+ 1.8%

*CT* computed tomography.

*P* < 0.0167 was considered to be statistically significant, with Bonferroni correction for multiple comparisons.

### Associations between airway volumes on CT in standing, sitting, and supine positions and measurements on PFT

Spearman’s coefficients (ρ) for the correlation between airway volumes on CT and measurements on the PFT are shown in Table 4. The coefficients for the correlation between the inspiratory airway volumes on CT and the measurements on the PFT were numerically higher in the standing position than in the supine position, with regard to vital capacity (0.681 vs. 0.660), forced vital capacity (0.673 vs. 0.659), forced expiratory volume in 1 s (0.669 vs. 0.643), inspiratory capacity (0.640 vs. 0.632), residual volume (0.558 vs. 0.546), functional residual capacity (0.607 vs. 0.585), and total lung capacity (0.726 vs. 0.709) (Table 4); however, no significant differences were found among these correlation coefficients between the standing and supine positions (all *P* > 0.0797). The coefficients for the correlation between the inspiratory airway volumes on CT and the measurements on the PFT were numerically higher in the sitting position than in the supine position, with regard to vital capacity (0.667 vs. 0.660), forced vital capacity (0.662 vs. 0.65), residual volume (0.573 vs. 0.546), functional residual capacity (0.629 vs. 0.585), and total lung capacity (0.721 vs. 0.709) (Table 4); however, no significant differences were found among these correlation coefficients between the sitting and supine positions (all *P* > 0.0938). The correlation coefficients between the inspiratory airway volumes on CT and the measurements on the PFT were numerically higher in the supine position than in the sitting position, with regard to forced expiratory volume in 1 s (0.643 vs. 0.641) and inspiratory capacity (0.632 vs. 0.604) (Table 4); however, no significant differences were found in these two correlation coefficients between the supine and sitting positions (all *P* > 0.3922).

**Table 4:** Spearman’s correlation coefficients between airway volumes on CT and measurements on PFT.

	Inspiratory airway volume in standing position		Inspiratory airway volume in sitting position		Inspiratory airway volume in supine position		Expiratory airway volume in standing position		Expiratory airway volume in sitting position		Expiratory airway volume in supine position	
	ρ	P value	ρ	P value	ρ	P value	ρ	P value	ρ	P value	ρ	P value
VC (mL)	0.681	< 0.0001	0.667	< 0.0001	0.660	< 0.0001	0.695	< 0.0001	0.698	< 0.0001	0.654	< 0.0001
FVC (mL)	0.673	< 0.0001	0.662	< 0.0001	0.659	< 0.0001	0.689	< 0.0001	0.692	< 0.0001	0.654	< 0.0001
FEV1 (mL)	0.669	< 0.0001	0.641	< 0.0001	0.643	< 0.0001	0.672	< 0.0001	0.672	< 0.0001	0.617	< 0.0001
Inspiratory capacity (mL)	0.640	< 0.0001	0.604	< 0.0001	0.632	< 0.0001	0.596	< 0.0001	0.593	< 0.0001	0.537	< 0.0001
Residual volume (mL)	0.558	< 0.0001	0.573	< 0.0001	0.546	< 0.0001	0.695	< 0.0001	0.671	< 0.0001	0.716	< 0.0001
Functional residual capacity (mL)	0.607	< 0.0001	0.629	< 0.0001	0.585	< 0.0001	0.744	< 0.0001	0.748	< 0.0001	0.760	< 0.0001
Total lung capacity (mL)	0.726	< 0.0001	0.721	< 0.0001	0.709	< 0.0001	0.785	< 0.0001	0.780	< 0.0001	0.760	< 0.0001

*CT* computed tomography, *PFT* pulmonary function test, *VC* vital capacity, *FVC* forced vital capacity, *FEV1* forced expiratory volume in 1 s.

## Discussion

To the best of our knowledge, this is the first study to show the differences in the airway volumes on CT among standing, sitting, and supine positions. Our study showed that the inspiratory and expiratory airway volumes as well as luminal areas of the trachea, bilateral main bronchi, and average third-generation airway were significantly higher in the sitting and standing positions than in the supine position. These findings are noteworthy because the results could provide important clues regarding the pathogenesis of orthopnea. In patients with chronic obstructive pulmonary disease (COPD), breathing discomfort can become amplified in the supine position (i.e. orthopnea)^17,18^. However, the precise mechanisms of orthopnea are still unknown^18^. Eltayara et al. reported that increased airway resistance in the supine position due to a lower end-expiratory lung volume probably plays a role in the genesis of orthopnea^17^. Considering our results, the increased airway resistance in the supine position due to a lower expiratory airway volume could also play a role in the development of orthopnea.

One possible reason for the difference in airway volume and luminal area between the upright (sitting and standing positions) and supine positions would be due to the difference in the direction of gravity. Several previous studies have reported that gravity affects the airway and chest^19–24^. Beaumont et al. reported that gravity affects the airway area and lung volume during parabolic flight using the acoustic reflection method and inductance plethysmography^19^. Elliott et al. evaluated the effect of spaceflight on sleep-disordered breathing and concluded that gravity plays an important role in the genesis of apneas, hypopneas, and snoring in healthy subjects^20^. In addition, it has been reported that gravity is associated with atelectasis^22^ and affects the chest wall mechanics^23,24^.

Our study also showed that the correlations between the inspiratory airway volume on CT and the measurements on the PFT tended to be higher in the standing position than in the supine position, although no statistically significant differences were found. This may be because PFTs are conducted in the upright position, and the direction of the thorax in PFTs corresponds to that in the upright CT rather than that in the conventional supine CT^3,4,25^. Furthermore, it is reported that the body position influences the results of PFTs^26–28^.

A previous study reported that the inspiratory airway luminal areas of the trachea, bilateral main bronchi, and average third-generation airway were larger in the standing than in the supine position^15^. Our results were, to some extent, consistent with these results; however, our study firstly evaluated inspiratory and expiratory airway luminal areas in the sitting position, the expiratory airway luminal areas in the upright position in an asymptomatic volunteer cohort, and the volume of the whole airway with lumen diameters of more than approximately 1.5 mm, which would be more reflective of the overall condition of the airway. Another previous study reported that the airway volume in the right upper and middle-lower lobes on conventional supine CT were correlated with the forced expiratory volume in 1 s in patients with COPD (correlation coefficient, 0.41)^14^; however, the authors assessed only the airway volume in the right lung and did not assess the left airway. We believe that measuring only the right airway may not allow overall lung function to be assessed. Actually, the correlation coefficient between the airway volume (whole bilateral airway) in the standing position and the forced expiratory volume in 1 s in this study was 0.669, relatively higher than that in the previous study (0.41)^14^. Whole airway volume in the standing position could be used as a new clinical indicator to evaluate the therapeutic effect or disease severity, and future studies investigating the correlation between airway volumes on upright CT and clinical findings in patient cohorts are needed.

The current study had some limitations. First, we included only 100 asymptomatic participants at a single institution, and further studies with large samples sizes at multiple centers are required to confirm these preliminary findings. Second, in this study, although the radiologists evaluated the CT images in a blinded and randomized manner, they could recognize, to some extent, the positions of the participants because of the presence or absence of a CT scanner table. However, the airway volume measurements were automated by using a commercially available workstation; thus, observer bias is considered to be negligible^4^. Third, conventional supine CT was performed with the arms raised in this study, whereas upright (standing and sitting) CT was performed with the arms down; thus, the form of the chest would have been slightly different between the upright and supine positions, which may have influenced the results of this study. However, we believe that standing or sitting with the arms down is the natural standing or sitting posture for human beings.

## Conclusions

The inspiratory and expiratory airway volumes and luminal areas of the trachea, bilateral main bronchi, and average third-generation airway were significantly higher in the sitting and standing positions than in the supine position, which could provide important clues regarding the pathogenesis of orthopnea. The airway volumes on both upright and conventional supine CT were moderately correlated with the PFT measurements.

## Data Availability

The datasets generated during and/or analyzed during the current study are available from the corresponding author on reasonable request.
